# Prevalence of ACTN3 (the athlete gene) R577X polymorphism in Iranian population

**Published:** 2012-10-30

**Authors:** Z Fattahi, H Najmabadi

**Affiliations:** 1 Genetics Research Centre, University of Social Welfare and Rehabilitation Sciences, Tehran, Iran

**Keywords:** Sport Genetics, ACTN3, Iran

## Abstract

**Background:**

Ability of athletes in speed or endurance contests somehow is determined by inherited muscle fiber types. One of the important genes involved in sport genetics is ACTN3 that is located on chromosome 11q13-q14 and encodes α-actinin-3, which belongs to highly conserved family of α-actinin proteins. Genetic analysis of α-actinin-3 gene has showed a polymorphism R577X (rs1815739), which results in premature stop codon and leads to non functional α-actnin-3 protein. ACTN3 genotype can contribute to the performance in elite and endurance activities.

R577X polymorphism replaces arginine by stop codon. Individuals homozygous for R577 have full copy of α-actinin-3 and elite and power sprint athletes show significantly higher frequency of 577R allele. In the other hand, some studies represented that X allele have high level of frequency in endurance athletes. However, this data remains controversial Since there is no information about the frequency of ACTN3 genotype in our population therefore as the first step it is essential to determine the genetic background of Iranian population. The objective of this study was to genotype normal Iranian individuals to determine the prevalence of each allele in our population.

**Methods:**

We used PCR-RFLP method for genotyping 210 normal individuals.

**Results:**

Total of 210 Iranian normal individuals for distribution of R577X and R alleles were genotyped. The different genotypes were as follow; 24% RR (50/210), 65%RX (136/210) and 11%XX (24/210), with allelic distribution of 0.56 and 0.44 for 577R and 577X alleles of ACTN3.

**Conclusion:**

This allelic distribution for Iranian's is more close to Caucasian population, which is concurrent with the route of ancient human's migration from Iran Plateau toward Europe.

Our results showed no different patterns of allelic distribution among female and males, which was the same in other studies too, although some differences has been reported in the studies on athletes population.

## Introduction

Ability of athletes in speed or endurance contests is somehow determined by inherited muscle fiber types.

Muscle fibers are multi-nuclei cells, responsible for muscle contractions. Each muscle is nearly composed of 10,000 to 450,000 muscle fibers.

There are two types of muscle fibers which are detectable on the color.Red muscle fibers (fiber type I or slow-ontracting fibers), due to high storage of blood and high percentage of myoglobin and mitochondria, can be observed in red. They slowly become exhausted and use the glycogen and fat as their fuel. White muscle fibers (type II fibers or fibers with fast contraction) have average blood storage and low levels of myoglobin and mitochondria. They only use glycogen as their fuel, and comparing to red fibers become tired more quickly. However, they are larger than red fibers with stronger contractions.

Red muscle fibers are consistent for long period aerobic activities and white muscle fibers for speed and short term anaerobic activities.

On average, these two types of contractile fibers are present equally in individuals. However, endurance athletes, have a greater percentage of red fibers (fibers with slow contraction) while, sprint athlete's have more white fibers (fast contracting fibers).

Distribution of these fibers in human muscles is also determined by genetic factors. One of the important genes involved in sport genetics is ACTN.(1)

ACTN3 is located on chromosome 11q13-q14 and encodes α-actinin-3 protein, which belongs to highly conserved family of α-actinin proteins.

This family contains cytoskeletal proteins and belongs to the Spectrin superfamily; α-actinin-3 is one of the sarcomeric actinin isoforms.

Indeed; there are two genes that encode skeletal-muscle α-actinins; ACTN2 (MIM 102573) and ACTN3 (MIM 102574).

These two proteins have different patterns of expressions, ACTN2 is expressed in all fibers (including all skeletal muscle fibers, cardiac muscles and also brain) whereas expression of ACTN3 is confined to fast (type 2) muscle fibers and produces higher amounts of force in fast movements. Low expression of ACTN3 has reported in brain, as well.

Both of these proteins are components of the Z-line and play an actin anchoring role in the muscle. However, it has been stated that these proteins have also regulatory functions in coordinating myofiber contractions and a role in maintaining muscle cell integrity by linking Dystrophin.(2, 3, 4, and 5)

Genetic analysis of α-actinin-3 gene has showed a polymorphism R577X (rs1815739), which results in premature stop codon in the gene and leads to non functional α-actnin-3 protein.

Although, at first it was considered responsible for some muscular diseases, but more investigation cleared this polymorphism as a common one in normal population.(6, 7)

This data, suggested that α-actinin-2 can compensate absence of α–actinin-3 in homozygous people for null allele of ACTN3. However, no up regulation of α-actinin-2 in the absence of α-actinin-3 and highly conservation of α-actinin-3 since its divergence from α-actinin-2 and additionally their different patterns of expression, all together suggests a distinctive role for this protein.(4)

Further researches showed that, ACTN3 genotype can be related to the performance in elite and endurance activities.

R577X polymorphism occurs in exon 16, which replaces Arginine by stop codon. Individuals homozygous for R577 have full copy of α-actinin-3 and elite and power sprint athletes show significantly higher frequency of 577R allele. In the other hand, some studies represented that X allele have high level of frequency in endurance athletes.(2, 4)

The mutated allele, 577X, has different frequencies in different populations which are shown in Table 1

**Table 1:** Frequencies of 577X and 577R alleles in different populations.^2^, ^8^

Population	Allele frequency of 577X	Allele frequency of 577R
**Asian**	0.50	0.50
**Javanese**	0.54	0.46
**Asian-Americans**	0.52	0.48
**Native American**	0.43	0.57
**Hispanic**	0.41	0.59
**European White**	0.42	0.58
**North Indian**	0.48	0.52
**Iranian**	0.44	0.56
**Aboriginal Australian**	0.29	0.71
**African American**	0.27	0.73
**African**	0.16	0.84
**African Bantu**	0.10	0.90

In ancient times, Iran was presumed in the way of human's migration, since there was no information about the frequency of ACTN3 in Iranian population, it seemed necessary to obtain information about genetic background of Iranian population. The objective of this study was to genotype normal Iranian individuals to determine the prevalence of each allele in our population.

## Materials and Methods

### Subjects

Subjects were 210 normal populations from different regions of Iran, including 110 female and 100 male. Distribution of different ethnic groups in Iranian population (considered in WHO) are as follow ; Fars 51% - Azeri 24% - Gilaki & Mazandarani 8% - Kord 7% - Arab 3% - Lor 2% - Baloochi 2% - Torkaman 2%.The sample size was calculated according to the formula [ss=Z ^2^^*^ P ^*^ (1-P) /C ^2^ ] with almost 93% confidence level ;and the samples were chosen in order to represent the distribution of different ethnic groups in Iranian population.

The Genetics Research Center at the University of Social Welfare and Rehabilitation Sciences, Tehran, Iran, approved all procedures.

### Genotyping

Genomic DNA was isolated from peripheral blood samples according to a standard procedure.(9)

Same as the other studies, RFLP-PCR method was chosen in the experiments. Primers used for amplifying were forward 5’- CTGTTGCCTGTGGTAAGTGGG -3’ and reverse 5’-TGGTCACAGTATGCAGGAGGG -3’.(2) 

The PCR reaction ingredients for the total volume of 30µl were as follow; Buffer 10X(withoutMgCl2):2.5µl, MgCl2 (50mM):0.75µl, dNTP(10mM); 0.5µl, Spermidin =0.025 µl, Taq polymerase(50U/µl):0.2µl, forward and reverse primers(0.1µM)=1 for each , DNA(50ng/µl)=1 and H2O=23.025 µl.

PCR reactions were optimized as following conditions; initial denaturation (95°C for 4min), denaturation (94°C for 40sec), annealing (60°C for 30sec) ,extension (72°C for 40sec) followed by final extension at 72°C for 2 min. PCR products (290 bp) were then digested by Dde1 (New England Biolab) at 37°C overnight and then analyzed by conventional Polyacrylamide gel electrophoresis (8%) followed by silver staining.

**Figure 1:**
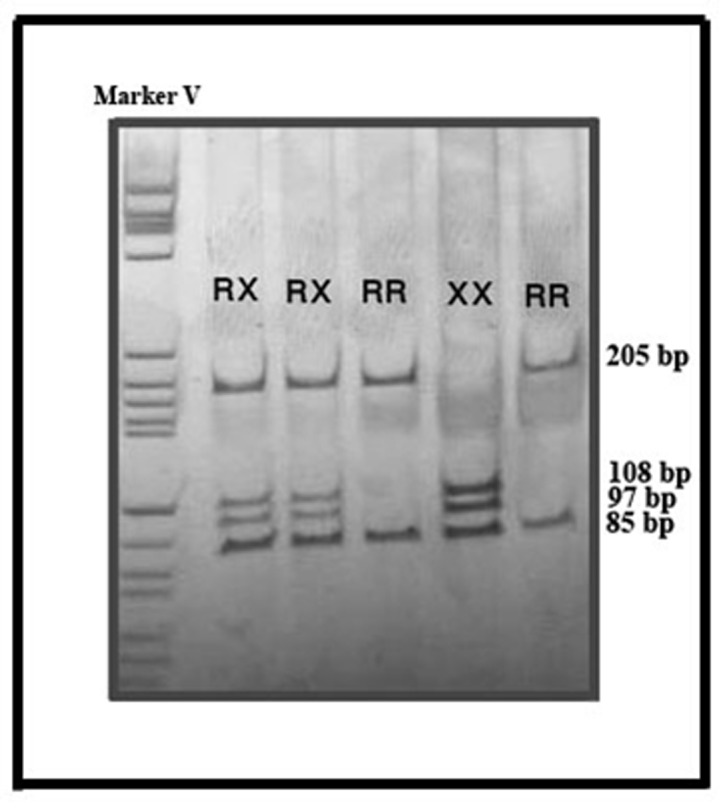
PCR-RFLP results on poly acryl amide gel (8%).RR; homozygote genotype for R577, shows two bands (205 and 85 bp) after digestion by Dde1.XX; homozygote genotype for X577, whit three bands (85, 97 and 108 bp). RX, heterozygote genotype, distinguished by 4 bands (85, 97,108 and 205bp).

Dde1 has two restriction sites on X allele and one site on R allele. So, PAGE results show two bands (205 and 85 bp) for RR genotype and three bands (85, 97 and 108 bp) for XX genotype (Figure1).

## Results

Genotyping of 210 Iranian normal individuals for distribution of RR, RX and XX genotypes of ACTN3 gene showed percentages of 24%, 65% and 11%, which leads us to the allelic distribution of 0.56 and 0.44 for 577R and 577X alleles of ACTN3 in our cohort of Iranian population. These frequencies have been calculated for female and male separately, which is shown in Table 2.

**Table 2:** Genotypes of ACTN3 and allele frequencies of X and R alleles in Iranian population

	Genotype frequency			Allele frequency	
	**RR**	**RX**	**XX**	**R**	**X**
**Female**	(30/110)	(67/110)	(13/110)	**0.58**	**0.42**
	27.27%	60.91%	11.82%		
**Male**	(20/100)	(69/100)	(11/100)	**0.545**	**0.455**
	20%	69%	11%		
**Total**	(50/210)	(136/210)	(24/210)	**0.56**	**0.44**
	23.81%	64.76%	11.42%		

## Discussion

It has been suggested that different frequencies of 577X allele in different populations is due to the relatively population-specific positive selection.(3)

As it can be observed in Table 1 the allele frequency of 577X differs from 0.5 in Asian people to about 0.16 in African people.(8)

Regarding to ancient human's migration; we expected the obtained results in this study.

As it has been mentioned, the allelic distributions of 0.56 and 0.44 for 577R and 577X alleles of ACTN3 gene in Iranian population is similar to Caucasian and also north Indian population, which is concurrent with the human's migration pathway.

The distinctive frequency of 577X suggests it as an ancient allele. So, during migration of human, its frequency has been changed based on different environmental conditions and natural selection.(3)

Our results showed no different patterns of allelic distribution among female and males, the same in other studies. Although some differences has been reported in the studies on athlete’s population. In the other hand, Iran is composed of different ethnic groups, sometimes with completely different genetic backgrounds. In this study, we tried to select samples from different parts of Iran randomly to have an overview of Iranian population. However; we are aware that population specific studies of our different ethnic groups may show different results.

Different studies have proposed that 577R allele has higher frequency in elite sprint and power athletes or in the other way, ACTN3 XX genotype is lower comparing to normal population.(4, 10, 11, 12, 13, 14) However, the role of 577X allele in endurance activities is still controversial.(10, 15)

Regarding to our similar genetic distribution of ACTN3 alleles comparing to Caucasians in normal individuals, it seems necessary to estimate this allelic distribution in Iranian athlete population, too. Especially because of our completely different championship results, it seems that we should pay more attention to genetic background of our athletes in addition of environmental factors (such as nutrition, workout etc.).

Our results show that distribution of these two alleles is in Hardy-Weinberg equilibrium in Iranian normal individuals.

Genetic tests should be involved in finding sport talents during childhood. This genetic test can involve ACTN3 gene, however it is noteworthy to mention that other genes are involved in sport genetics too.

Finding heritable sport talents of children followed by good environmental conditions can be a very effective way to train more world champions in our country.

## References

[1] Vincent B, Bock KD, Ramaekers M, Van den Eede E, Van Leemputte M, Hespel P, Thomis MA (2007). ACTN3 (R577X) genotype is associated with fiber type distribution.. Physiol Genomics.

[2] Goel H, Mittal B (2007). ACTN3: Athlete gene prevalence in North India.. Current science.

[3] MacArthur DG, North KN (2004). A gene for speed? The evolution and function of alpha-actinin-3.. Bioessays.

[4] Yang N, MacArthur DG, Gulbin JP, Hahn AG, Beggs AH, Easteal S, North K (2003). ACTN3 genotype is associated with human elite athletic performance.. Am J Hum Genet.

[5] Yang N, Garton F, North K (2009). α-Actinin-3 and Performance.. Med Sport Sci.

[6] North KN, Yang N, Wattanasirichaigoon D, Mills M, Easteal S, Beggs AH (1999). A common nonsense mutation results in a-actinin-3 deficiency in the general population.. Nat Genet.

[7] Suminaga R, Matsuo M, Takeshima Y, Nakamura H, Wada H (2000). Nonsense mutation of the alpha-actinin-3 gene is not associated with dystrophinopathy.. Am J Med Genet.

[8] Mills M, Yang N, Weinberger R, Vander Woude DL, Beggs AH, Easteal S, North K (2001). Differential expression of the actin-binding proteins, alpha-actinin-2 and -3, in different species: implications for the evolution of functional redundancy.. Hum Mol Genet.

[9] Miller S.A, Dykes D.F (1988). a simple salting out procedure for extracting DNA from human nucleated cells.. Nucleic Acids Research.

[10] Alfred T, Ben-Shlomo Y, Cooper R, Hardy R, Cooper C, Deary IJ, Gunnell D, Harris SE, Kumari M, Martin RM, Moran CN, Pitsiladis YP, Ring SM, Sayer AA, Smith GD, Starr JM, Kuh D, Day IN (2011). The HALCyon study team.ACTN3 genotype, athletic status and lifecourse physical capability: meta-analysis of the published literature and findings from nine studies.. Hum Mutat.

[11] Chiu LL, Wu YF, Tang MT, Yu HC, Hsieh LL, Hsieh SS (2011). ACTN3 Genotype and Swimming Performance in Taiwan.. Int J Sports Med.

[12] Druzhevskaya AM, Ahmetov II, Astratenkova IV, Rogozkin VA (2008). Association of the ACTN3 R577X polymorphism with power athlete status in Russians.. Eur J Appl Physiol.

[13] Massidda M, Vona G, Calò CM (2009). Association between the ACTN3 R577X polymorphism and artistic gymnastic performance in Italy.. Genet Test Mol Biomarkers.

[14] Roth SM, Walsh S, Liu D, Metter EJ, Ferrucci L, Hurley BF (2008). The ACTN3 R577X nonsense allele is under-represented in elite-level strength athletes.. Eur J Hum Genet.

[15] Ahmetov II, Druzhevskaya AM, Astratenkova IV, Popov DV, Vinogradova OL, Rogozkin VA (2008). The ACTN3 R577X polymorphism in Russian endurance athletes.. Br J Sports Med.

